# A tale of two meta‐analyses: Similarity, consistency and avoiding overinterpretation in network meta‐analysis

**DOI:** 10.1111/jdv.70367

**Published:** 2026-03-25

**Authors:** J. Shourick, P. Garbayo‐Salmons

**Affiliations:** ^1^ Service d'Epidémiologie, Clinique et de Santé Publique CHU de Toulouse, Pôle Santé Publique et Médecine Sociale Toulouse France; ^2^ Department of Dermatology, Parc Taulí Hospital Universitari, Institut d'Investigació i Innovació Parc Taulí (I3PT‐CERCA) Universitat Autònoma de Barcelona Sabadell Spain

Network meta‐analysis (NMA) were introduced by Lumley et al^1^ in 2004 and have since exploded in medical sciences. They are powerful tools, used to examine a multiplicity of treatments in a particular situation and help rank them in terms of efficacy and safety.

Like all meta‐analyses, NMA rests on the *homogeneity* assumption, which means that studies comparing the same treatments are comparable.^1^ For instance, in hidradenitis suppurativa (HS), this implies that the phase III trials SUNRISE and SUNSHINE, which both compared secukinumab to placebo, should have a similar evaluation of this comparison. Additionally, NMA also uses indirect evidence to estimate the effect of a treatment. This means that if treatment A is superior to treatment B by one point and B is superior to C by two points, then A must be superior to C by three points. The comparisons between A and B and between B and C are based on direct evidence, whereas the comparison between A and C relies on indirect evidence. This leads to the two next assumptions of NMA: *similarity*, which means that all comparisons should be conducted in sufficiently similar contexts so that homogeneity stands for the network as a whole and, *consistency*, which means that indirect and direct comparisons should lead to similar evidence.^1^ These three major assumptions have recently been summarized into the concept of *exchangeability*, which means that the assessed specific studies and the way they are performed are not important because they are all evaluating the same treatment effects in the same manner. However, although they might lead to the same final concept, increasing the similarity of the studies included may require excluding some studies that would provide direct evidence of key importance to evaluate consistency. Indeed, in a landscape of clinical trials overwhelmingly using placebo instead of active comparators, every study with an active comparator might prove very valuable.

Notably, when evaluating the comparative efficacy of the three biological treatments approved for HS: adalimumab, secukinumab and bimekizumab, Naik et al. and Calabrese et al. have used different methodological aproaches.^2^, ^3^ While Naik et al. have included all HS trials (phase 2, phase 3, extension studies and even the SHARPS trial, which had a very different context), Calabrese et al. only included phase 3 trials leading to an increased similarity of trials. Therefore, Calabrese et al. likely provide a better evaluation of each treatment effect, but with no opportunity to evaluate consistency. These two NMA lead to a different conclusion, one that bimekizumab was the best treatment (Naik et al.) and the other that adalimumab (Calabrese et al.) was the best treatment. It should be noted that the study by Naik et al. was funded by UCB, the manufacturer of bimekizumab and was not deposited on PROSPERO registry beforehand. However, despite this flaw and conflict of interest, the apparent divergence might be more tenuous than is apparent. Indeed, both meta‐analyses found very similar treatment effects for adalimumab and bimekizumab compared to placebo, which has been confirmed by a recent ongoing NMA of all medical interventions for HS (including the promising unpublished results of the phase 2 trial of sonelokimab), which found that bimekizumab had an OR of 0.84 95% CI [0.58, 1.35] of reaching HiSCR‐50 compared to adalimumab. Making both treatments very similar in terms of efficacy.^4^ However, Calabrese et al. also noted that adalimumab presented fewer adverse events than bimekizumab.

Finally, all phase 3 trials in HS have been performed against placebo. As already suggested in psoriasis, treatment efficacy hierarchization would be greatly simplified if regulatory agencies required the use of the best treatment available as a comparator rather than placebo.^5^

## FUNDING INFORMATION

The authors have nothing to report.

## CONFLICT OF INTEREST STATEMENT

P. Garbayo‐Salmons declares honoraria for participating in advisory boards from Novartis and UCB; received support for attending meetings and/or travel from AbbVie, Amgen, Lilly, LEO Pharma, Novartis and UCB. J. Shourick has nothing to disclose.

## ETHICS STATEMENT

Not applicable.

## Data Availability

Data sharing is not applicable to this article as no new data were created or analyzed in this study.
